# The potential role of nanomedicine in the treatment of breast cancer to overcome the obstacles of current therapies

**DOI:** 10.3389/fphar.2023.1143102

**Published:** 2023-02-22

**Authors:** Fan Yang, Qingjie He, Xiangpeng Dai, Xiaoling Zhang, Dong Song

**Affiliations:** ^1^ Breast Surgery Department of General Surgery, The First Hospital of Jilin University, Changchun, China; ^2^ Key Laboratory of Organ Regeneration and Transplantation of Ministry of Education, First Hospital of Jilin University, Changchun, China; ^3^ National-Local Joint Engineering Laboratory of Animal Models for Human Disease, First Hospital of Jilin University, Changchun, China

**Keywords:** nanomedicine, breast cancer, drug resistance, drug delivery, combined therapy

## Abstract

Breast cancer (BC) is the most frequently diagnosed malignant tumor among women in the world. BC is the heterogeneous tumor with different subtypes including luminal A-like, luminal B-like (HER2-/HER2+), HER2 enriched, and triple-negative BC. The therapeutic strategies including surgery, chemotherapy, radiotherapy, targeted therapy, and endocrine therapy are well developed and commonly used in the treatment of BC. However, some adverse effects of these conventional treatments limited their wide application in clinical. Therefore, it is necessary to develop more safe and more efficient individualized treatment strategies of the BC. Nanomedicine, as the most promising strategy for controlled and targeted drug delivery, is widely used in multiple aspects of cancer therapy. Importantly, accumulative evidences show that nanomedicine has achieved good outcomes in the treatment of BC and a huge amount of BC patients benefited from the nanomedicine related treatments. In this review, we summarized and discussed the major problems occurred during the administration of conventional treatment strategies for BC and the potential roles of nanomedicine in promoting the treatment efficacy of BC by overcoming obstacles of current treatment of BC.

## 1 Introduction

**FIGURE 1:**
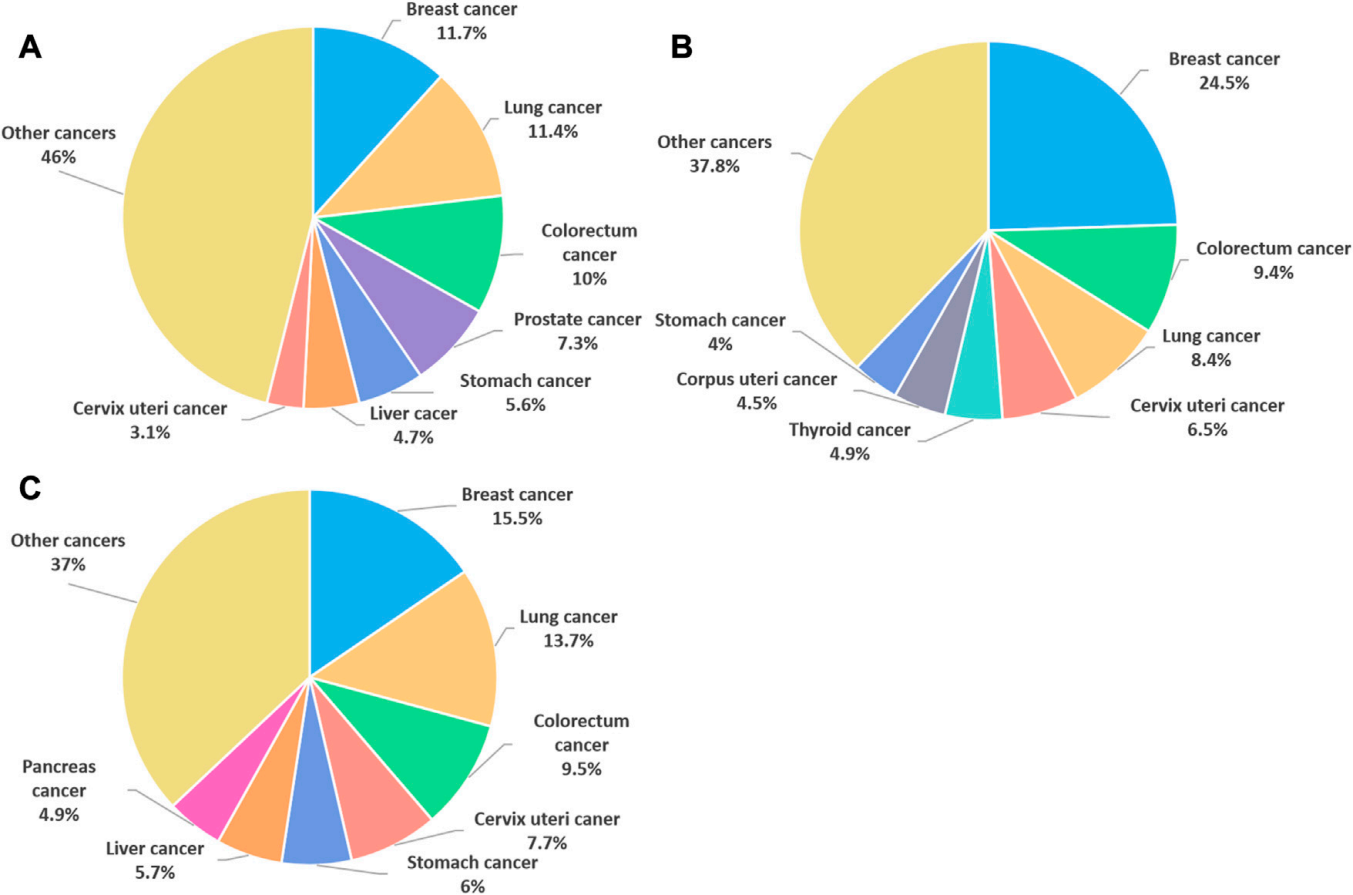
The cancer cases and deaths in 2020. **(A)** Percentage of new cancer cases worldwide in 2020, both sexes. **(B)** Percentage of new cancer cases worldwide in 2020, females. **(C)** Percentage of female cancer deaths worldwide in 2020. (data source: The World Health Organization).

**FIGURE 2:**
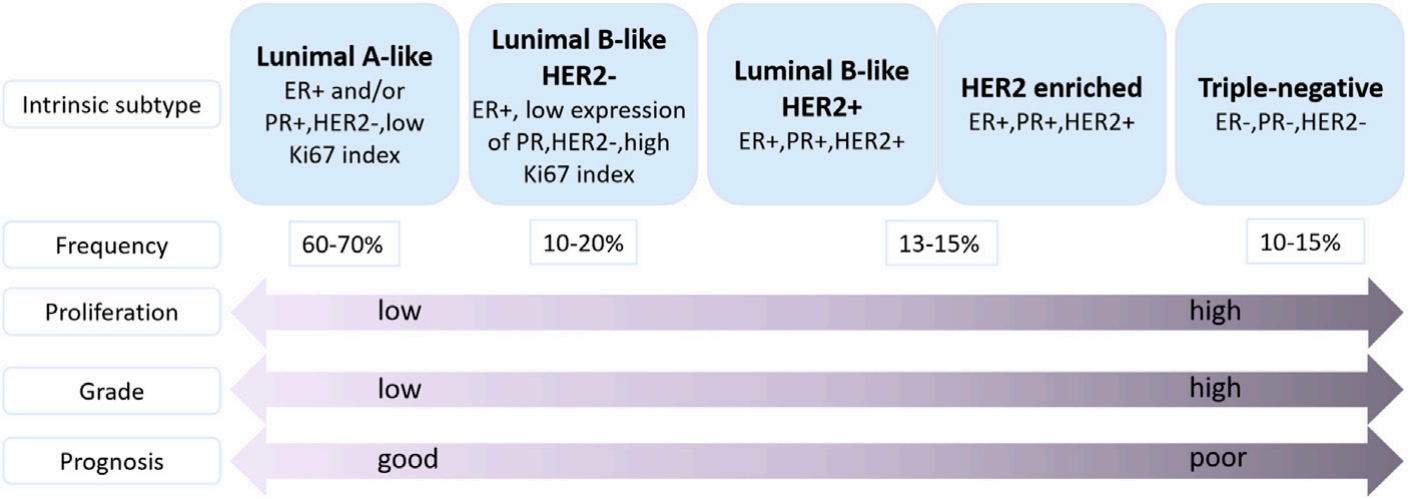
Classification of molecular subtypes of breast cancer (Harbeck et al., 2019). ER: estrogen receptor, PR, progesterone receptor; HER2, human epidermal growth factor receptor 2; Ki67, proliferation marker.

Breast cancer (BC) is the most commonly diagnosed cancer in female worldwide. There were about 2.3 million new cases of BC and the number of deaths reached 685,000 in 2020, accounting for 1 in 6 cancer deaths (Sung et al., 2021) (Figure 1). Moreover, BC is a heterogeneous cancer type and was classified into four main subtypes: luminal A, luminal B, human epidermal growth factor receptor 2 (HER2) enriched and basal-like BC. Currently, clinical relevant surrogate subtypes based on the histological and molecular characteristics included luminal A-like, luminal B-like HER2-, luminal B-like HER2+, HER2 enriched, and triple-negative type (Perou et al., 2000; Cheang et al., 2009; Harbeck et al., 2019) (Figure 2). Generally, multidisciplinary approaches which are used to treat BC include systemic therapy (chemotherapy, endocrine therapy, HER2 targeted therapy and so on) and local therapy (surgery and radiotherapy). The choose of therapeutic strategy depends on subtype and disease stage of BC (Waks and Winer, 2019; Shien and Iwata, 2020). Although the optimization of these treatment schemes has increased the cure opportunity of about 70%–80% of early BC patients, so far, metastatic BC is still considered as incurable (Harbeck et al., 2019). In addition, disadvantages of conventional anti-cancer drugs, such as lack of selectivity for tumors, poor solubility, high toxicity, multidrug resistance, short half-life and poor chemical stability, often lead to the therapeutic efficacy far from satisfactory (Fraguas-Sanchez et al., 2022). Hence, the more efficient and less toxic therapeutic strategies warrant further in deep investigation to develop personalized treatment for BC.

The inherent limitations of current used conventional anti-cancer treatments promoted the study of nanotechnology in cancer treatment. This nanotechnology often has higher efficacy and greater security, known as nanomedicine (Shi et al., 2017; Afzal et al., 2021). Compared with conventional chemotherapy, nanotechnology-based therapies exhibit distinct advantages: delivering drug to tumor sites *via* a passive or active targeting strategy, improving the solubility and chemical stability of drugs, prolonging the circulation time of drugs in the blood, reducing the antineoplastic-related toxicity and overcoming drug resistances mechanisms (Fraguas-Sanchez et al., 2022; Fu et al., 2022). With the rapid growth of nanotechnology in the past decades, nanomedicine has been widely used in basic research and clinical study, and has become highly promising and prevalent for cancer therapy. BC has become the focus of targeted nanomedicine research in various types of cancer, due to its heterogeneity, the high frequency of drug resistance, recurrence and chemotherapy failure (Jiang et al., 2022). For example, Abraxane^®^ was approved to treat metastatic BC in 2005 as regular therapeutics. This is a biologically interactive, nanoscale albumin-bound paclitaxel particle and research results indicated that compared with free PTX, this nanoscale albumin-bound paclitaxel has better curative effect and lower toxicity during the treatment of BC (Gradishar et al., 2005). In addition, other nanomedicines approved for clinical treatment of BC also exhibit great efficacy in suppressing BC progression (Table 1) (Boix-Montesinos et al., 2021). Moreover, nanomedicine can deliver a variety of therapeutic molecules to tumor sites *via* passive targeting, active targeting and stimuli responsive tumor targeting, such as chemotherapeutic drugs, photosensitizers, photothermal agents, therapeutic radioisotopes, immunotherapeutic adjuvants and immune checkpoint inhibitors. Hence, comprehensive anti-tumor strategies of nanomedicine therapy combined with chemotherapy, radiotherapy, hyperthermia, immunotherapy, etc. will provide promising therapeutic option (Liu W. et al., 2021).

**TABLE 1:** Approved nanomedicine for the treatment of breast cancer.

Product name	Nanocarrier	Drug	Approval organization and date
Doxil^®^/Caelyx^®^)	Liposome	Doxorubicin	FDA (1995), EMA (1996)
Myocet^®^	Liposome	Doxorubicin	EMA (2000)
Abraxane^®^	Albumin	Paclitaxel	FDA (2005, 2012, 2013)
			EMA (2008)
Lipusu^®^	Liposome	Paclitaxel	FDA (2006)
Nanoxel^®^	Polymeric micelles	Paclitaxel	FDA (2006)
Genexol^®^	Polymeric micelles	Paclitaxel	FDA (2007)
Lipodox^®^	Liposome	Doxorubicin	FDA (2013)
Kadcyla^®^	Antibody	Trastuzumab/DM1	FDA (2013)

The nano medicines composed of various nanoparticles were loaded with chemotherapeutic drugs exhibit great therapeutic effect on the treatment of breast cancer.

In this review, we carefully summarized and discussed the main problems occurred during the administration of conventional treatment strategies for BC and the potential roles of nanomedicine in promoting the treatment efficacy of BC by overcoming obstacles of current treatment of BC. Furthermore, the combination therapies of current used therapeutics of BC with nanomedicine are also highlighted.

## 2 The current used therapeutic strategies for BC

### 2.1 Molecular basis of distinct BC subtypes

BC is a proliferative type of carcinoma originating from the breast tissue (Gupta et al., 2021). Molecularly, BC is classified as luminal A-like, luminal B-like (HER2-/HER2+), HER2 enriched, and triple-negative breast cancer (TNBC) (Figure 2), based on the presence or absence of distinct molecular: estrogen receptor (ER), progesterone receptor (PR), HER2 and the proliferation marker Ki67 which classification is commonly used in clinical practice (Gupta et al., 2021; Banthia et al., 2022). Luminal A-like subtype is ER and/or PR positive, HER2 negative, and low Ki67 index, suggesting a better prognosis compared with other subtypes (Gao and Swain, 2018). Luminal B-like (HER2-) tumors are ER and/or PR positive, HER2 negative, and high Ki67 index or low expression of PR. Luminal B-like (HER2+) tumors are ER positive, HER2 positive with any PR level, and any Ki67 level (Gao and Swain, 2018; Hashmi et al., 2018). HER2 enriched tumors are defined as HER2 positive, ER and PR negative (Schettini and Prat, 2021). Triple-negative subtype is a basal-like subtype of BC which is ER negative, PR negative and HER2 negative (Derakhshan and Reis-Filho, 2022). Because of the lack of recognized therapeutic molecular targets, the prognosis of TNBC patients is often worse than other types of BC patients. Furthermore, the TNBC often occurs in younger women (Malorni et al., 2012; Bergin and Loi, 2019).

The occurrence of BC is usually highly associated with genetic factors and environmental factors. It was reported that the levels of estrogen and androgen are positively correlated with the risk of BC (Endogenous et al., 2013). Furthermore, genetic mutations of BRCA1, BRCA2, TP53, CDH1, PTEN, STK11, ATM, BRIP1, PALB2, CHEK2 and NBS1 are known to be closely related to the increased risk of developing BC (Gupta et al., 2021; Lukasiewicz et al., 2021).

### 2.2 Current common treatment strategies of BC

The subtypes, stage and grade of BC, the age, physical condition and medical condition of patients are important factors which should be paid attention to when making a decision to choose a suitable treatment method. For non-metastatic BC, the main treatment purpose is to eliminate the tumor and prevent metastasis and recurrence. And for metastatic BC, the treatment goal is to reduce symptoms and prolong life of patients (Waks and Winer, 2019). In general, the common therapeutic strategy of BC includes systemic treatment and local treatment.

#### 2.2.1 Systemic treatment of BC

##### 2.2.1.1 Chemotherapy

Chemotherapy is the most extensively applied systemic treatment strategy for BC. The main anticancer mechanism of most conventional cytotoxic chemotherapeutic drugs is to suppress the rapid division and growth of cancer cells (Omidi et al., 2022), which typically results in cancer cell death by targeting the cancer cells at different cell cycle stages (Chabner and Roberts, 2005). Currently, commonly administered agents in the chemotherapy of BC are anthracyclines (doxorubicin, epirubicin), taxanes (PTX, docetaxel), platinum agents (cisplatin, carboplatin), cyclophosphamide, and so on (Saloustros et al., 2008; Penel et al., 2012; Yamaguchi et al., 2015; Garutti et al., 2019). These chemotherapeutic agents can exert anti-tumor effects by oral, intravenous or intrathecal injection (Zagouri et al., 2013). In addition, it was found that multidrug combination therapy can generally improve the anti-tumor effect which cannot be achieved by the administration of single chemotherapy drug (Junnuthula et al., 2022). Besides, chemotherapy includes adjuvant chemotherapy and neoadjuvant chemotherapy. Adjuvant chemotherapy is the chemotherapy administered after surgery for BC patients with lymphatic metastases or high risk of suffering a recurrence (Early Breast Cancer Trialists' Collaborative et al., 2012). While chemotherapy applied to patients before operation is called neoadjuvant chemotherapy which determines the response of tumor to chemotherapy, lowers the tumor stage, and increases patient eligibility for breast conservation surgery, and it has important clinical value for locally advanced and inoperable BC (Wang and Mao, 2020; Davey et al., 2021; Sun et al., 2022).

##### 2.2.1.2 Endocrine therapy

Endocrine therapy is the main treatment method for hormone receptor positive (ER positive and/or PR positive) BC patients. And this treatment can be used as a neoadjuvant therapy or adjuvant therapy for patients with Luminal A or Luminal B subtype of BC (Zelnak and O'Regan, 2015; Lerebours et al., 2021). The goal of endocrine therapy is to block the function of estrogen or lower the level of estrogen which could stimulate the growth of BC cells. The drugs for endocrine therapy mainly include the selective estrogen receptor modulators (SERMs) (tamoxifen, toremifene), selective estrogen receptor degraders (SERDs) (fulvestrant) and aromatase inhibitors (AIs) (letrozole, anastrazole, exemestane) (Lumachi et al., 2011; Reinbolt et al., 2015).

##### 2.2.1.3 HER2 targeted therapy

The main characteristic of HER2 enriched subtype is the overexpression of HER2, which displays more rapid tumor growth, more aggressive development, and is related to worse survival results compared with the Luminal A and B subtypes (Boix-Montesinos et al., 2021). Therefore, HER2 targeted therapy is essential for patients diagnosed with the HER2-enriched subtypes. At present, the main drugs for HER2 targeted therapy include monoclonal antibodies (trastuzumab and pertuzumab), tyrosine kinase inhibitors (neratinib, lapatinib, etc.), and antibody-drug conjugate such as trastuzumab-emtansine (T-DM1) (Bredin et al., 2020).

##### 2.2.1.4 Immunotherapy

Immunotherapy is also a systemic treatment regime for BC and it can prevent, regulate and eliminate BC cells by inducing the patients’ natural defense system (Ahmad et al., 2022; Fraguas-Sanchez et al., 2022). The internal mechanism of immunotherapy is strengthening the immune system to specifically identify and destroy malignant cells (Navarro-Ocon et al., 2022). In addition, immunotherapeutic agents can not only treat primary tumor, but also prevent distant metastasis and reduce recurrence rate (Gavas et al., 2021). For example, patients with TNBC are more likely to benefit from immunotherapy than those with other types of BC due to the existence of mutations, tumor-infiltrating lymphocytes (TILs) and increased levels of programmed death ligand 1 (PD-L1) expression (Kwapisz, 2021). And atezolizumab, a PD-L1 inhibitor, was approved to be combined with nab-paclitaxel in the treatment of locally advanced or metastatic TNBC patients whose tumors express PD-L1 (Kwapisz, 2021).

#### 2.2.2 Local treatment and local therapy of BC

##### 2.2.2.1 Surgery

Mastectomy and lumpectomy (also called breast-conserving surgery) are two main types of surgical operations for different stages of BC (Sharma et al., 2010). And studies showed that mastectomy and lumpectomy followed by radiotherapy are equivalent in terms of recurrence and overall survival (Fisher et al., 2002). Moreover, the pathological status of axillary lymph nodes in BC patients is a significant prognostic indicator. Compared with sentinel lymph node biopsy (SLNB), axillary lymph node dissection (ALND) often leads to more serious postoperative complications, such as paresthesia and lymphedema (Li et al., 2015). Therefore, SLNB, a definitive method to exclude axillary metastases, has supplanted ALND as the main method to evaluate the axilla in most patients with early BC (Ecanow et al., 2013).

##### 2.2.2.2 Radiotherapy

Radiation therapy is a treatment that utilize high energy radiation to destroy cancer cells, and it has been used for treatment of different cancers for over a century. The effect of radiation on tumor cells was originally discovered by treating a woman with locally advanced BC, and now the radiation has played a core role in the treatment of BC (Ye and Formenti, 2018). Radiation therapy mainly includes two kinds of treatments: external beam radiotherapy (EBRT) and internal radioisotope therapy (RIT) (Liu W. et al., 2021). Radiation therapy can be applied to the breast after breast-conserving surgery, chest wall after mastectomy, and regional lymph nodes (Boyages, 2017) to ensure that cancerous cells are destroyed, and reduce cancer recurrence chances.

## 3 Obstacles of current treatment strategies of BC

With the development of technologies related to cancer treatment, standard therapeutic strategies of BC have indeed made great progress, which have surely decreased the mortality rate, and enabled most patients to recover from cancer (Lee et al., 2017). Nevertheless, there are still some obstacles during the treatment of BC by current therapeutic strategies.

It was well known that the chemotherapy is the primary therapeutic option for BC, but conventional treatments of chemotherapy still have several significant drawbacks. Firstly, the distribution of chemotherapeutic agents is lack of the specificity for tumors. These chemotherapeutic agents suppress the rapid division and growth of cancer cells as well as the proliferation of normal cells of the body owing to non-specific tumor targeting, which result in the inevitable adverse effects (Omidi et al., 2022), such as alopecia, nausea, vomiting, diarrhea, mouth ulcers, tiredness, increased susceptibility to infections, myelosuppression, combined with leucopenia, anemia, easier bruising or bleeding (Lukasiewicz et al., 2021). In addition, other drug-specific side effects were also reported such as anthracyclines-induced cardiotoxicity and cisplatin-induced-ototoxicity and nephrotoxicity (Dilruba and Kalayda, 2016; Saleh et al., 2021). Secondly, drug resistance is another obstacle of conventional chemotherapeutic agents, which reducing the efficacy of drug treatment in cancer cell. The drug resistance could be divided into intrinsic resistance and acquired resistance according to the time of occurrence (Wang et al., 2019). The fundamental mechanisms of chemoresistance are extremely complicated and include increased efflux of drugs, tumor heterogeneity, enhanced DNA damage repair, epigenetic alterations, cell death inhibition (apoptosis suppression), alteration of drug target, inactivation of the anticancer drugs, changes in drug metabolism and tumor microenvironment (Mansoori et al., 2017; Wang et al., 2019). Notably, drug efflux transporters which is found to be responsible for the drug efflux, and efflux pumps, are mainly correlated with the process of multidrug resistance of tumor. Efflux transporters belong to the ATP-binding cassette (ABC) transporter superfamily, and human genome embodies 48 ABC genes which are divided into seven subfamilies (ABCA-ABCG) (Gote et al., 2021). It was reported that the P-glycoprotein (P-gp/MDR1/ABCB1), multidrug resistance-associated protein 1 (MRP1/ABCC1) and breast cancer resistance protein (BCRP/ABCG2) are the major ABC transporters correlated with multidrug resistance in BC (Rizwanullah et al., 2021). Thirdly, poor solubility and high toxicity of chemotherapeutic drugs also affect the therapeutic effect of chemotherapy. A majority of chemotherapy drugs derived from plant source or synthesis are hydrophobic and require solvents to formulate the dosage, which increase the toxicity of drug preparations and limit the dosage (Chidambaram et al., 2011). Finally, short half-life and poor chemical stability also compromise the therapeutic efficacy of chemotherapy drugs, affecting the delivery and absorption rate in the tumor site and hindering the dose-effect (Malik et al., 2022). Furthermore, most chemotherapy drugs cannot cross the blood brain barrier (BBB), which limits the therapeutic effect of BC brain metastases (Mills et al., 2020). Unfortunately, 30% of early-stage BC have recurrent disease, and most of them are metastases (Burguin et al., 2021).

The most common adverse events of endocrine therapy are hot flashes and night sweats, vaginal dryness, increased risk of thromboembolic events, bone-related adverse events, such as osteoporosis (Condorelli and Vaz-Luis, 2018). And drug resistance to hormone therapies is also a challenge faced by BC treatment (AlFakeeh and Brezden-Masley, 2018). Although trastuzumab and pertuzumab have showed positive effects on the treatment of HER2-enriched BC, intrinsic resistance and acquired resistance are common event during treatment which warrant an in deep understanding of underlying drug resistance mechanisms to guide the research and development of novel HER2 targeted drugs (Rimawi et al., 2015).

Similarly, drug resistance is also a major therapeutic obstacle in immunotherapy. Besides, another major weak point of immunotherapy, particularly in a combined therapy, is the occurrence of immune related side effects causing various adverse reactions in skin and gastrointestinal such as rash, pruritus, diarrhea and colitis (Garcia-Aranda and Redondo, 2019). Apart from the risk of relapse, as a short-term solution, surgery also leads to long-term adverse effects such as anatomical changes, chronic pain, phantom breast pain, lymphedema and so on (Lovelace et al., 2019). Consistently, the radiotherapy can cause radiation dermatitis, radiation pneumonia, myelosuppression, cardiac and pulmonary injury, radiation-induced malignancy, fatigue, swelling and lymphedema and other side effects, interfering with activities of daily living (Lovelace et al., 2019; Sun et al., 2022).

In consideration of the aforementioned obstacles of conventional treatments, challenges of BC treatment include overcoming multidrug resistance and recurrence, and alleviating or avoiding the side effects brought by treatment. Therefore, it is vital to develop novel therapy methods for effective treatment of BC, addressing the unmet medical need faced by BC patients.

## 4 Nanomedicine promotes the treatment efficiency of BC by overcoming the obstacles of current therapeutics of BC

Recent years, the nanomedicine exhibited a number of advantages in helping to overcome the obstacles of traditional treatments of BC. Nanotechnology enables operation in materials with a size from 1 to 100 nm at least one dimension (Banthia et al., 2022). For example, the nanoparticles (NPs) which are technologically defined as particles smaller than 100 nm with one dimension, were mainly designed for targeted drug delivery (Boisseau and Loubaton, 2011). The NPs not only improve the biological distribution of drugs, targets active molecules to diseased tissues, but also protects healthy tissues by avoiding the distribution of drugs in normal tissues (Boisseau and Loubaton, 2011). The unique properties of NPs include the small size, large surface-to-volume ratio, adjustable physical and chemical properties, ability to load large amounts of drugs, longer circulation time, high uptake and retention, tumor-targeting efficacy, sustained release of the chemotherapeutic payload, biocompatibility, bioavailability, increased circulation time and overcoming multidrug resistance (Banthia et al., 2022). Moreover, the compact scale of nanoparticle allows them to break through biological barriers, such as the BBB (Tagde et al., 2022), providing an opportunity for the treatment of BC patients with brain metastases.

### 4.1 Synthetic methods of NPs

It was well known that the NPs have different shapes, sizes and structures. Hence, a variety of synthesis methods are adopted, roughly dividing into two categories: bottom-up method and top-down method. For the bottom-up method, the raw materials are miniaturized beforehand at the molecular level or atomic level, after that self-assemble into NPs, or additional catalytic agents are added to help them assemble (Tagde et al., 2022). While for the top-down method, the non-materials needed are made of external and macroscopic raw materials, and this method well-controls the processing of these macroscopic materials from the outside (Tagde et al., 2022). That is to say, a larger molecule is broken down or decomposed into smaller units, and then converted to NPs.

### 4.2 Classification of NPs used in medicine

**FIGURE 3:**
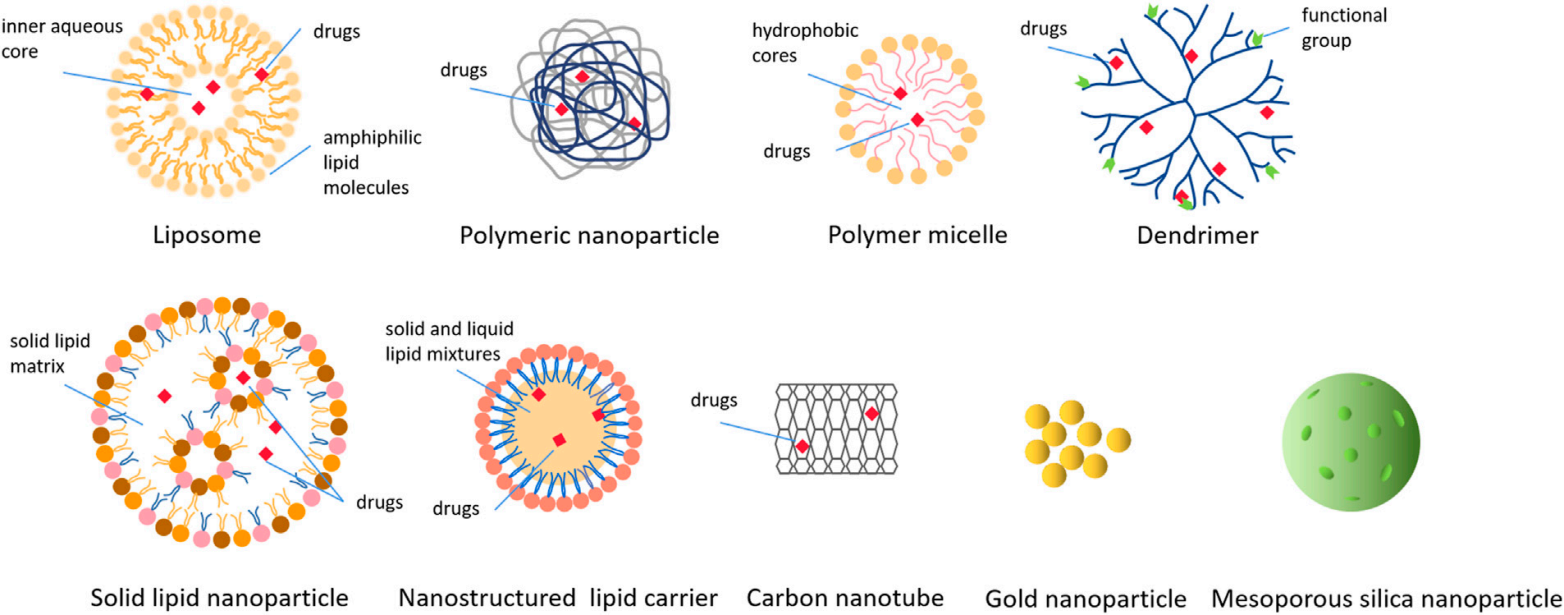
Various types of nanoparticles used to deliver drugs in cancer treatment. These nanoparticles include organic nanoparticles and inorganic nanoparticles, and this picture shows the structure and drugs loading of various nanoparticles in detail.

Currently, a large number of NPs, with different sizes, shapes, surface charge, microstructure and surface modification (Figure 3), are used as carriers to deliver the payload in the treatment of human diseases. These NPs include organic NPs (such as liposomes, polymeric nanoparticles, polymeric micelles, dendrimers, etc.) and inorganic NPs (such as carbon nanotubes, metallic nanoparticles, quantum dots, etc.). Here, we summarized several NPs involved in the development of nanomedicines to treat BC.

#### 4.2.1 Liposomes

Liposomes are spherical nanovesicles formed by amphiphilic lipid molecules (Alavi and Hamidi, 2019). One side of such lipid molecules is hydrophilic and the other side is hydrophobic. Hence, this characteristic enables they to form spherical particles containing an inner aqueous core immediately when combined with water (Alavi and Hamidi, 2019). Typically, liposomes range in size from 25nm to 2.5 μm with one or more bilayer membranes (Saravanakumar et al., 2022). This unique property of liposomes enables them to effectively load hydrophobic drugs in the lipid bilayer and encapsulate hydrophilic drugs in the internal aqueous core. Moreover, the cell membrane-like structure makes it easy for the liposomes to fuse with the cell membrane and thus deliver the loaded drugs to the cell (Jiang et al., 2022). Importantly, liposomes were the first nanomedicines tested in FDA clinical trials (Bobo et al., 2016). Because the immune system can recognize the lipid bilayer structures of liposomes, and macrophages can clear these lipid bilayer structures from the circulation, conventional liposomes have short circulating half-lives (Ventola, 2017). However, in liposomal NPs, this kind of clearance can be minimized by PEGylation in the liposome surface (Ventola, 2017). Thus, the blood circulation time of liposomal NPs is longer, providing better therapeutic effect for patients. For example, Doxil^®^, the first Food and Drug Administration (FDA)-approved nanomedicine in 1995, is the PEGylated liposome loaded with doxorubicin (DOX) hydrochloride, which can be applied to the treatment of metastatic BC, and it reduces systemic toxicity, maintains the antitumor properties of DOX and increases the circulation time avoiding premature elimination (Barenholz, 2012). Until now, several nanomedicines have been approved by FDA for the treatment of BC (Table 1). Furthermore, liposomes can be also used as co-delivery systems to deliver chemotherapeutic agents and inhibitors, making cancer cells sensitive to anticancer drugs. Tang *et al.* found that co-encapsulated DOX and verapamil liposome could not only overcome P-gp-mediated multidrug resistance of BC cells, but also reduce the toxicity of important non-target organs (Tang et al., 2016).

#### 4.2.2 Polymeric nanoparticles

Polymeric nanoparticles (P-NPs) are synthesized from biodegradable and biocompatible raw materials (polyesters) and the P-NPs can load chemotherapy drugs by encapsulation or conjugation (Afzal et al., 2021). Nevertheless, controlling the molecular weight, polydispersity and stereoregularity of polymers is the main disadvantage of these methods (Juan et al., 2020). Hence, at present, polyesters such as polycaprolactone (PCL) and polylactide (PLA) are often prepared by ring-opening polymerization of lactone or lactide rings under the action of organometallic catalysts (Tang et al., 2017; Juan et al., 2020). P-NPs could be exploited utilizing naturally-occurring biocompatible polymers, that are comprised of ester, amide, ether and other functional groups (Juan et al., 2020). The PCL, PLA and poly (lactic-co-glycolic acid) (PLGA) are biocompatible polymers approved by FDA to be used in drug delivery and other polymers such as polyethylene glycol (PEG), chitosan (CS) and hyaluronic acid (HA) are used for drug delivery as well (Saravanakumar et al., 2022). Additionally, by adjusting the properties of the polymers or modifying the surface with various ligands, P-NPs target and control drugs release, thus improving the bioavailability and treatment effect (Zielinska et al., 2020).

#### 4.2.3 Polymeric micelles

Polymer micelles are nanocarriers obtained by self-assembly of hydrophilic shells and hydrophobic cores (Yadav et al., 2022). Most hydrophilic shells are composed of PEG, poly (glutamic acid) (PGA), poly (ethyleneimine) (PEI), etc., while hydrophobic cores are composed of PCL, poly (lactic acid) (PLA), PLGA (Avramovic et al., 2020). With average particle size of 5–100 nm (Alven and Aderibigbe, 2020), polymer micelles can enhance the penetration of tumor vascular system, making it a very effective drug carrier (Hanafy et al., 2018). Besides, hydrophobic core has the advantage of retaining hydrophobic drugs in the core (Yadav et al., 2022), and they are usually widely used to distribute anti-cancer drugs with low water solubility, such as PTX. Peng et al. (2019) constructed a kind of worm-like nanocrystal micelles composed of Herceptin conjugated PTX loaded PCL-PEG, which are used to treat HER2-positive BC, and the study showed that the PTXs remain stable in circulation and tumor microenvironment, and have specific HER2+tumor cell targeting, sparing normal tissues from the toxic effects.

#### 4.2.4 Dendrimers

The dendritic nanoparticle is composed of three parts, a central core, repeating branching units and outer surface functional group, with a size ranging from 1–100 nm (Kesharwani et al., 2022). Dendrimers are especially helpful for improving the solubility of hydrophobic drugs, because the core of dendrimers is usually hydrophobic (Saravanakumar et al., 2022). The branched structures of dendrimers have various modifications to choose from, so that high drug content and targeted drug delivery can be better achieved (Jain et al., 2010). Besides, the outer surface functional group can be extensively chemically modified or complexed with drugs, targeting ligands, as well as imaging agents (Kim et al., 2018). The dendrimers widely used in cancer treatment include poly (L-lysine) (PLL) dendrimers, polypropylene imine (PPI) dendrimers, polyamidoamine (PAMAM) dendrimers, and PAMAM-organosilicon dendrimers (PAMAMOS) (Kesharwani et al., 2022). Therefore, these diversified characteristics and properties make dendrimers a good platform for drugs delivery in the treatment of cancers. For example, Guo et al. (2019) synthesized a novel type of PAMAM dendrimer nanoparticles modified by HA for co-delivery of cisplatin and doxorubicin (HA@PAMAM-Pt-Dox). Study results showed that the HA@PAMAM-Pt-Dox can effectively kill breast cancer cells and exhibited a high potency in improving the chemotherapy efficacy of cisplatin and DOX.

#### 4.2.5 Solid lipid nanoparticles (SLNs)

Solid lipid nanoparticles (SLNs) are composed of solid lipid matrix, and this matrix is characterized by its solid state at room temperature and body temperature. The solid lipids in SLNs contain long-chain fatty acids, fatty acid esters, and waxes (Tagde et al., 2022). Drugs delivery is achieved by embedding into lipid core or binding to the lipid surface. SLNs are the most common method to improve the bioavailability of oral drugs with poor water solubility, and they exhibit a great deal of advantages, such as easy manufacture, the stability of drugs, increased drug load, effective drug release and high long-term stability (Afzal et al., 2021).

#### 4.2.6 Nanostructured lipid carriers (NLCs)

Although SLNs have the above advantages, problems such as limited drug loading, drug leakage crystallization during storage and unexpected polymorphic transitions (Behravan et al., 2022) need to be better solved. Fortunately, the other type of lipid NPs, nanostructured lipid carriers (NLCs), can overcome these shortcomings of SLNs (Banthia et al., 2022). NLCs are composed of solid and liquid lipid mixtures. As the second generation of lipid based nanocarriers, NLCs have an unstructured matrix because of the different components of NLCs (Haider et al., 2020). Although NLCs remain solid state in nature, even at body temperature, their melting points are lower than SLNs. Moreover, NLCs in liquid form provide more space for drug dissolution and payload due to their unstructured properties and defects in crystallization behavior (Tagde et al., 2022).

#### 4.2.7 Carbon nanotubes

The diameter of carbon nanotubes is in the nanometer range, which are cylinders composed of one or more coaxial graphite layers (Sargent et al., 2009). Owing to intrinsic hydrophobicity, drugs can be encapsulated in carbon nanoparticles *via* π—π stacking (Ou et al., 2016). Carbon nanotubes can accommodate high payload because of considerable surface area, and they have unique optical, electronic emission and mechanical properties (Afzal et al., 2021). Therefore, carbon nanotubes can be used as instruments for the distribution and release of targeting and regulating drugs, as contrast agents for diagnosis and recognition of breast tumors, as well as biosensors (Hashemi et al., 2017).

#### 4.2.8 Gold nanoparticles (AuNPs)

Gold nanoparticles (AuNPs) have many advantages, including their biocompatibility, multifunction, high photothermal conversion efficiency, imaging contrast ratio and easy modified surfaces. Therefore, AuNPs are excellent photothermal therapy (PTT) agents (Granja et al., 2021). Due to the high efficiency of photothermal conversion, AuNPs can convert the light energy in near-infrared region (NIR) into heat energy, thus killing cancer cells (Li et al., 2016).

#### 4.2.9 Mesoporous silica nanoparticles (MSNs)

Mesoporous silica nanoparticles (MSNs) have become drugs delivery carriers because of the large surface area, adjustable pore size and release properties, high drug content capacity, zero premature release and versatile capabilities (Poonia et al., 2018). Hence, MSNs are regarded as one of the best drug carriers owing to their better pharmacokinetic properties.

#### 4.2.10 Quantum dots (QDs)

Quantum dots (QDs) are the nanometer-scale semiconductors with the size between 2 and 10 nm (Nitheesh et al., 2021), composed of a core of crystalline metalloids and the shell (Tharkar et al., 2015). QDs have specific optical properties, broad excitation spectrum, and very narrow symmetrical intense distribution, which allow them to be used for bioimaging, biolabeling, and biosensing (Fatima et al., 2021). Dong and co-workers constructed a versatile ultrasmall Ag2Te QDs for high-performance computed tomography (CT) imaging-guided photonic tumor hyperthermia. Moreover because of the high photo-thermal conversion efficiency (50.5%), these Ag2Te QDs with negligible toxicity and excellent biocompatibility showed a high tumor inhibition rate (94.3%) on 4T1 cells in xenograft animal models (Dong et al., 2020).

### 4.3 Nanomedicine-based therapeutic strategies of BC

Nanomedicine-based therapeutic strategies targeting tumor can be broadly divided into passive targeting, active targeting and stimuli responsive tumor targeting.

#### 4.3.1 Passive targeting

Passive targeting is the result of the enhanced permeability and retention (EPR) effect, which leads to the accumulation of NPs in tumor tissues because of the leakage of vasculature in the tumor microenvironment (Prabhakar et al., 2013). Usually, rapidly growing tumor cells respond to hypoxia situations through the process of neovascularization. Compared with normal vessels, the new blood vessels often have large pores, which lead to the permeability selectivity of tumor vessels worse (Torchilin, 2011). Besides, in normal tissues, extracellular fluid keeps constant drainage and renewal through lymphatic vessels. The formation of tumor leads to lymphatic dysfunction and then the interstitial fluid absorption is minimal (Gavas et al., 2021). This is another feature of EPR which contributes to the retention of NPs in the tumor (Ernsting et al., 2013). This effect is the key to obtain selective tumor accumulation of chemotherapy drugs for BC. Nevertheless, human tumors are heterogeneous in many aspects, including pore distribution, hypoxia area, pericellular coverage, basement membrane and extracellular matrix, which will reduce the effectiveness of EPR targeting cancer cells (Jiang et al., 2022).

#### 4.3.2 Active targeting

The surface of tumor vascular endothelial cells usually abnormally express specific antigens or receptors when tumor tissues grow rapidly, while the surface of blood vessel cells in normal tissues rarely or even not express those specific antigens or receptors (Wei et al., 2021). Hence, surface modification of NPs with corresponding antibodies or ligands can increase their enrichment in tumor tissues and endow them the ability of targeted delivery of drugs (Wei et al., 2021). The important mechanism of this active targeting is recognition of ligands by target substrate receptors. And the ligands generally include peptides, proteins, nucleic acids, antibodies, sugars, and small molecules like folic acid (Byrne et al., 2008). Additionally, the choice of ligands depends on the overexpression of targets in each cancer subtype (Alibakhshi et al., 2017). For instance, in HER2-enriched BC, HER2 is the most commonly used target because they are involved in the progression of this subtype of tumor. Hence, the use of monoclonal antibodies against HER2 receptor is a good active targeted therapy (Fraguas-Sanchez et al., 2019). Clinically, in TNBC, antibody-drug conjugates that target trophoblast cell-surface antigen 2 (Trop-2) and NMB glycoproteins, zinc transporter LIV-1, protein tyrosine kinase 7 (PTK7) receptor, and ephrin receptor-4 through active targeting therapy have also been studied (Fraguas-Sanchez et al., 2022). In addition, the active targeting sites involve tumor vasculatures, tumor stroma, tumor cells, immune cells (Jiang et al., 2022) and the subcellular level (Gupta et al., 2021) due to the modification of different target ligands.

#### 4.3.3 Stimuli responsive tumor targeting

Stimuli responsive tumor targeting refers to the release of payload by triggering NPs at cancer tissues due to internal or external stimuli, which increases its efficacy and decreases its systemic toxicity by this selective release of the drug at tumor sites (Fraguas-Sanchez et al., 2019). The internal stimuli contain changes in redox potential, enzymes, and pH (Oshiro-Junior et al., 2020). While external stimuli include temperature, photodynamic therapy, ultrasound, electric field, and so on (Gote et al., 2021). Li et al. (2021) designed an instant pH-responsive size-shrinkable drug delivery system named self-aggregated DOX@HA-CD (SA-DOX@HA-CD), which loaded with DOX and prepared with small size HA modified carbon dots (HA-CD) as monomers. SA-DOX@HA-CD could self-aggregate into raspberry-like structure in neutral pH and represent good blood compatibility and stability. However, in the simulated tumor microenvironment (pH 6.5), it could rapidly decompose into shotgun-like CD monomer loaded with DOX due to the changes in charge, hydrophilicity and hydrophobicity, enhancing cellular uptake and deep penetration in breast cancer model and effectively improving the efficacy of chemotherapy (Li et al., 2021).

### 4.4 Nanoparticle plays important roles in the combined therapies for BC

Currently, the monotherapy is usually found to be difficult to obtain satisfactory anti-cancer effect. Compared with monotherapy, combined therapy with multiple strategies can produce synergistic anti-cancer effect and reduce the adverse effects caused by individual drug treatment. Hence, the nanoparticle mediated combination of chemotherapy and other therapies for BC was summarized and discussed in this section.

#### 4.4.1 Photodynamic therapy (PDT) in combination with chemotherapy for BC

Photodynamic therapy (PDT) involves delivering photosensitizer (PS) through local or other systemic options, and then irradiates targeted tissues with light of a specific wavelength, which is suitable for the given PS (Aghajanzadeh et al., 2022). And that photosensitizers do not accumulate in the nuclei, thereby preventing them from causing cancer (Aghajanzadeh et al., 2022). Chemotherapy combined with PDT is one of the common methods to enhance the anti-cancer effect. For example, Liu X. L. et al. (2021) constructed a liposomal system, nano-Pt/VP@MLipo, that loaded Platinum nanoparticles (nano-Pt), and verteporfin (VP), a clinical PS, is loaded in the lipid bilayer that endow PDT activity. This liposomes biomimetic nano-Pt/VP@MLipo effectively targeted the tumor regions, in which the oxygen generated by nano-Pt catalyzation improved the PDT mediated by VP, and PDT in turn made the liposome membrane permeable, so as to effectively release nano-Pt and enhance the chemotherapy effect (Liu X. L. et al., 2021). In another study, Pan et al. (2022) constructed a cancer-targeted nano-platform (PFTT@CM), composed of Fe^3+^, tetrakis (4-carboxyphenyl) porphyrin (TCPP) and hypoxia-activable prodrug tirapazamine (TPZ). The Fe3+ of PFTT@CM triggered ferroptosis, reduced the GSH and generated •OH along with oxygen, then the TCPP and light-mediated PDT procedure would use up oxygen and exacerbated tumor hypoxia, thus further activating the prodrug TPZ for cancer chemotherapy.

#### 4.4.2 Photothermal therapy (PTT) in combination with chemotherapy for BC

Photothermal therapy (PTT) has attracted extensive interest owing to its minimal invasiveness and particular spatial and temporal selectivity (Yang et al., 2022). Chemotherapy combined with PTT has become a promising therapeutic strategy for BC. For example, Shen et al. (2019) designed a PLGA-based therapeutic nanoplatform (IDPNs) to jointly deliver the widely applied near-infrared dye indocyanine green (ICG) and chemotherapeutic drug DOX. The IDPNs showed superior stability, photothermal effect, biocompatibility, and on-demand drug release behavior. The chemo-photothermal combination therapy resulted in a preferential chemical-photothermal combination treatment effect *in vitro* and efficiently suppressed tumor growth in nude mice bearing with BC cells without apparent systemic toxicity.

#### 4.4.3 Chemodynamic therapy (CDT) in combination with chemotherapy for BC

Chemodynamic therapy (CDT) is an efficient method for cancer treatment that can produce highly cytotoxic hydroxyl radicals (⋅OH), leading to serious oxidative damage and cell death. The CDT required neither oxygen nor external energy input, but relied on the Fenton catalysts (Xin et al., 2022). The advantages of CDT include high tumor specificity and selectivity, low systemic toxicity, and less side effects. Moreover, chemotherapy in combination with CDT can reduce the side effects of chemotherapy drugs, and improve the efficacy of CDT. Xue et al. (2019) synthesized a nanoparticle termed DMH NPs, that consisted of MIL-100, which could serve as a nanocarrier to load DOX, and HA modified on the surface of MIL-100. At the same time, MIL-100 could produce OH through Fenton-like reaction in the presence of H_2_O_2_ for CDT. Research indicated that the chemotherapy in combination with CDT could effectively induce the MCF-7 cells death, enhance antitumor efficacy and reduce drug-related toxicity (Xue et al., 2019).

#### 4.4.4 Immunotherapy in combination with chemotherapy for BC

Currently, immunotherapy in combination with chemotherapy based on nanocarrier-based drug delivery systems is also a promising approach for BC therapy. Liu et al. (2018) designed a dual pH-responsive versatile nanoparticle system comprised of R848 (immune-regulator) encapsulated with poly (L-histidine) (PHIS), and DOX (chemotherapeutics) conjugated to HA. The ionization of PHIS near pH 6.5 (pH value is close to that of tumor microenvironment) changed the property of this material from hydrophobic to hydrophilic, then triggered the release of R848 to play an immunomodulatory role, and hydrazone bond in HA-DOX broke at around pH 5.5 (pH of endo/lysosomes), which speeded up the release of DOX to play the role of chemotherapy (Liu et al., 2018). Animal experiment showed that HA-DOX/PHIS/R848 NPs had strong tumor targeting ability in 4T1 tumor-bearing mice, because of EPR and CD44-mediated active targeting, as well as significantly inhibited tumor growth through regulating tumor immunity and killing tumor cells (Liu et al., 2018).

## 5 Conclusion and outlook

In recent decades, the application of nanomedicine in the treatment of BC has made significant progress. The nanomedicine can lower the toxicity and overcome chemoresistance of conventional chemotherapy by passive targeting, active targeting and stimuli responsive tumor targeting of nanocarriers to tumor cells. Various types of NPs, such as liposomes, polymeric nanoparticles, polymeric micelles, dendrimers, carbon nanotubes, etc. have been explored and employed for targeted drug delivery. This article overviewed the current obstacles of conventional treatments of BC and the prospects of nanotherapeutics for BC therapy. Despite nanomedicines have showed a good application prospect in the treatment of BC disease, there are still several problems which need to be addressed before the nanomedicine was used in the clinical practice, such as long-term toxicity of nanomaterials, their impact on the immune system, pharmaceutical stability issues reproduction of uniform NPs batches and so on. In short, nanomedicine is an effective method to treat BC alone or in combined with other therapeutic strategies, and it is necessary to further study and develop the more safe and efficient therapeutics based on nanotechnology for cancer treatment.
